# Artificial intelligence (AI)-based multi-organ contour quality assurance with uncertainty estimation for online adaptive radiotherapy (oART)

**DOI:** 10.1088/3049-477X/ae3320

**Published:** 2026-01-19

**Authors:** Shunyu Yan, Jiacheng Xie, Na Chen, Dan Nguyen, Fan-Chi Su, Daniel Yang, You Zhang, Steve Jiang, Chenyang Shen

**Affiliations:** The Medical Artificial Intelligence and Automation (MAIA) Laboratory and Department of Radiation Oncology, University of Texas Southwestern Medical Center, Dallas, TX 75390, United States of America

**Keywords:** contour quality assurance, artificial intelligence, uncertainty quantification, online adaptive radiotherapy, deep learning

## Abstract

Accurate delineation of treatment targets and organs at risk (OARs) is essential to the success of radiotherapy (RT). Although artificial intelligence (AI)–based segmentation methods have successfully automated the delineation process, a reliable and efficient quality assurance (QA) mechanism is still missing, particularly in the time-critical setting of online adaptive RT (oART). This study aims to address this unmet clinical need by introducing an AI-driven multi-organ contour QA framework with uncertainty quantification. Using MR-guided oART for prostate cancer as the testbed, we developed an automatic multi-structure QA framework by employing a carefully designed contour quality estimation (ConQuE) model. Built on a ResNet34 backbone, ConQuE processes binary segmentation masks with corresponding MR images to classify contours as ‘acceptable’ or ‘revision required’ in a slice-by-slice manner. To ensure scalability, we proposed a unified framework for multi-organ QA by embedding structure-specific code into the fully connected layers. To quantify classification uncertainty, Monte Carlo (MC) dropout was employed, enabling confidence assessment of model predictions for informative decision-making in oART. ConQuE was trained to assess the segmentation quality of prostate and six OARs, including rectum, bladder, urethra, penile bulb, cauda equina, and femoral heads. Training was performed using 249 cases for training, 31 for validation, and another 31 cases were saved for testing. With 30 MC dropout passes, ConQuE can complete QA for one image slice in ∼13.1 ms. Overall, ConQuE achieved accuracy of 93.9% on all structures with structure-specific performance consistently above 90%, demonstrating its strong capability in identifying *revision required* contours. Moreover, incorrect predictions were strongly correlated with high uncertainty scores, underscoring the effectiveness in improving QA reliability. The proposed AI-based framework holds the potential to automate contour QA with quantified uncertainty. It is well-suited for oART with heavy time constraints given its high accuracy, efficiency, and reliability.

## 1. Introduction

Accurate delineation of treatment targets and organs-at-risk (OARs) is essential for radiotherapy (RT) planning, as it plays a critical role in plan optimization and assessment, ultimately impacting patient safety and treatment efficacy [1]. However, conventional segmentation by clinicians is often time-consuming, labor-intensive, and prone to inter-observer variability and human errors [2]. In recent years, artificial intelligence (AI)-based automatic segmentation models [3–7] have gained increasing attention for their potential to streamline the delineation process and reduce the workload of clinicians. These models have demonstrated encouraging results in segmentation consistency and efficiency compared to conventional methods in many settings [8, 9]. However, despite promising results, AI-based segmentation models also face several limitations. Firstly, these models often struggle to accurately delineate complex anatomical sites or disease regions with inherently low image contrast [10–12], resulting in inconsistent segmentation performance across organs. Additionally, training and clinical data are inherently inhomogeneous due to variations in imaging protocols, which may lead to model performance degradation over time when applied across diverse clinical datasets [13]. Moreover, these AI-based segmentation models are often regarded as black boxes, lacking reliable confidence measures making it difficult to assess the trustworthiness of the model outputs, limiting interpretability and clinical adoption in safety-critical scenarios [14]. Given the existing limitations of current AI-based segmentation tools, segmentation errors remain inevitable in RT workflows. Undetected segmentation errors can substantially affect treatment planning accuracy, compromising patient safety, and leading to suboptimal treatment quality and outcomes.

Given these limitations, there is a critical need for reliable and efficient quality assurance (QA) mechanisms to identify potential segmentation errors and support safe clinical deployment of AI-generated contours. Currently, the standard clinical practice for contour QA relies on manual review of segmented contours conducted by experienced clinicians. This approach is usually labor-intensive, time-consuming, and often prone to human errors. Especially for time-sensitive workflows such as online adaptive RT (oART), manual contour QA substantially extends the patient’s on-couch time. This not only deteriorates the ART plan quality due to patient motion and further anatomical changes while waiting, but also increases patient discomfort and anxiety, potentially compromising the treatment quality and outcome. Therefore, there is a pressing clinical need for efficient and reliable QA tools capable of identifying suboptimal contours automatically.

Recently, both knowledge-driven and learning-based approaches have been developed to automate the evaluation of segmentation accuracy and identify suboptimal contours. Knowledge-driven QA methods typically rely on geometric feature analysis [15, 16], image texture analysis [17], and statistical outlier detection [18–20] to identify abnormal contours. However, these methods are often limited by their reliance on predefined geometric and spatial features. For instance, contours that are clinically unacceptable may still fall within acceptable metric thresholds if their shape or position appears reasonable. More recently, learning-based methods, particularly deep-learning models have emerged as powerful tools that leverage data-driven approaches to automatically evaluate contour quality. Most current AI-based contour QA approaches assume a pre-trained segmentation model and build another model to predict geometric metrics such as Dice similarity coefficient or Hausdorff distance for the contour QA purpose [21–23]. Typically, they only produce definitive contour quality predictions without quantifying uncertainty or the confidence levels associated with these predictions. The lack of uncertainty quantification can lead to unreliable clinical decisions, especially in borderline cases, potentially resulting in inaccurate contours being adopted into treatment planning process [24]. Such inaccuracies may reduce the overall treatment efficacy and adversely impact patient safety.

To overcome these challenges, uncertainty estimation, which provides a quantitative measurement of model confidence, has been proposed to help identify cases where the model has less confidence about its predictions in many different applications, such as classification [25, 26], segmentation [27–30], dose prediction [31], and image generation tasks [32, 33]. In the context of segmentation, Monte Carlo (MC) dropout [27, 34] is one of the most commonly adopted approaches for estimating uncertainty, often integrated into segmentation models to generate uncertainty or probability maps [22, 27, 35–39]. For instance, Ozdemir *et al* [39] incorporated MC dropout into a lung cancer detection framework, where uncertainty maps generated from low-dose CT scans were used to guide bounding box extraction for malignancy prediction via an auxiliary classifier. Balagopal *et al* [27] proposed an anatomy-guided multi-task network for post-operative prostate segmentation, utilizing MC dropout to estimate voxel-level uncertainty and provide confidence bounds to support clinical review. Similarly, Min *et al* [35] also demonstrated the use of MC dropout in a QA framework based on a 3D ResUnet++, generating an average segmentation and an uncertainty band, followed by logistic regression for QA assessment. Despite its wide application in segmentation tasks, its use for quantifying the confidence of QA decisions remains largely unexplored. Additionally, current uncertainty-based QA methods are primarily designed for single-organ segmentation, with an inference time (e.g. ∼1.3 min per case) that may be acceptable for isolated use but become impractical when scaled to multi-organ QA in oART workflows with heavy time constraints.

To address the limitations of these existing contour QA methods, we propose an AI-driven multi-organ segmentation QA framework for efficient and reliable contour quality prediction. Using MR-guided oART (MRgOART) as the test bed, the proposed framework classifies the contours of multiple segmented structures as ‘*acceptable*’ or ‘*revision required*’ with MC dropout to provide a quantitative measurement of prediction confidence. By jointly estimating contour quality and associated confidence scores, the proposed strategy enhances interpretability of QA results and supports an informative decision-making process for the downstream contour editing. It also enables simultaneous evaluation of multiple anatomical structures using a single model, simplifying the workflow and significantly reducing the inference time.

## 2. Methods

### 2.1. Proposed workflow

**Figure 1.**
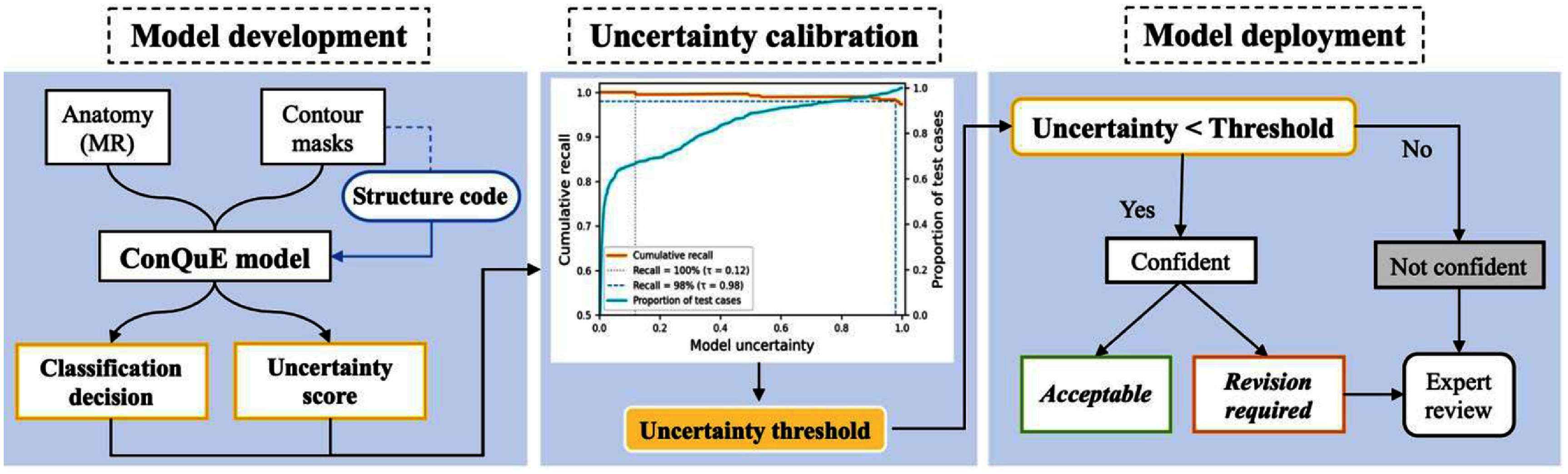
Proposed clinic AI-based contour QA system (ConQuE) workflow.

The overall goal of this framework is to not only flag potentially inaccurate segmentations but also identify cases with low prediction confidence, assisting clinicians to prioritize which contours to review and edit. To achieve this, we developed the contour quality estimation (ConQuE) model to assess segmentation quality across multiple organs and support automated decision-making. To provide clearer guidance and enhance clinical usability, we adopted a binary classification scheme that categorizes segmented contours as either ‘*acceptable’* or ‘*revision required*’, enabling a simple and actionable decision for clinicians in real-world clinical settings. As illustrated in figure 1, the workflow consists of three key stages: model development, uncertainty calibration, and model deployment. In the model development stage, the ConQuE model was trained to predict contour quality based on paired MR image, binary contour mask, and structure-specific code with an associated uncertainty score reflecting its confidence in predictions. Specifically, a fixed contour quality threshold was applied to the predicted quality score during training and evaluation to determine the classification labels and to assess prediction accuracy. We hypothesized that the prediction accuracy would exhibit an inverse correlation with the uncertainty score.

The goal of this calibration stage is to determine an uncertainty level threshold for the trained ConQuE model with validation data to predict contour quality reliably in real clinical applications. More specifically, we constructed a ‘recall vs. uncertainty’ calibration curve and derived an uncertainty threshold that satisfies a predefined recall rate for *revision required* cases desired in the clinic. Note that this calibration process can be performed again as needed using the data accumulated after model deployment to enhance its performance against potential drift in data.

In the model application stage, the determined uncertainty threshold is applied to guide the decision-making process. More specifically, predictions with uncertainty below this threshold are considered sufficiently reliable for automated decisions in contour quality, while those with uncertainty exceeding the threshold value will be routed to clinicians regardless of the predicted quality for further evaluation.

This framework ensures that only highly accurate predictions in contour quality are used for the automated QA process, while those unreliable predictions are flagged for further review by human experts. This approach is designed to ensure the effectiveness of contour QA while substantially improving overall QA efficiency by guiding the attention of clinicians to focus only where it is most needed.

### 2.2. Model design & training details

**Figure 2.**
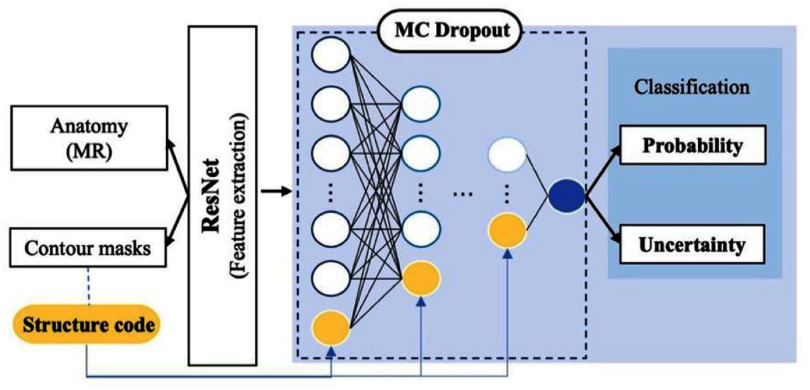
Network architecture of the proposed ConQuE model.

Using ResNet34 [40] as the backbone, the ConQuE framework was developed to evaluate the clinical acceptability of segmented contours in a slice-by-slice manner. A diagram of model architecture is depicted in figure 2. The model takes paired axial MR slice and corresponding binary contour masks as input to predict whether the contours are clinical acceptable or require modifications. Critical features for contour quality prediction are extracted for MRI image and contours, followed by a set of fully connected layers for the classification purpose where one-hot encoded structure representations are appended. To estimate prediction uncertainty, we implemented MC drop-out for all fully connected layers with a drop-out rate of 0.1.

The model was trained using a binary cross-entropy loss function with Adam optimizer. The initial learning rate was set as ${10^{ - 4}}$ while a learning rate scheduler was employed, reducing the rate by $80{\% }$ at every $100$ epochs. Each training epoch, we utilized a balanced number of *acceptable* and *revision required* samples for each organ. Training was performed using a batch size of $64$ on a single NVIDIA V100 GPU with 32 GB RAM.

### 2.3. Uncertainty estimation

Estimating uncertainty in deep learning models is challenging due to both the computational overhead of existing methods and the lack of streamlined approaches for clinical integration [26]. Traditional methods, such as deep ensembles [41], require training and maintaining multiple independently initialized models, which is computationally expensive and often infeasible in time-sensitive clinical workflows. An alternative approach is to adopt Bayesian inference [42], which estimates uncertainty by learning a distribution over the model weights $W$ given data $X$ and label $Y$. The Bayesian posterior $p\left( {W{\mathrm{|}}X,Y} \right)$ is defined as:

\begin{equation*}p\left( {W{\mathrm{|}}X,Y} \right) = \frac{{p\left( {Y{\mathrm{|}}X,W} \right)p\left( W \right)}}{{p\left(Y|X\right)}}\end{equation*}

where $p\left( W \right)$ is the prior distribution over the model weights, $p\left( {Y{\mathrm{|}}X,W} \right)$ is the likelihood of observing the data given the weights, and $p(Y|X)$ is the marginal likelihood that normalizes the distribution. And the corresponding predictive distribution is defined as an integral over the posterior distribution of model weights:

\begin{equation*}p\left( {y{\mathrm{|}}x,X,Y} \right) = \int {p\left( {y{\mathrm{|}}x,W} \right)} p\left( {W{\mathrm{|}}X,Y} \right){\mathrm{d}}W\end{equation*}

where $y$ is the predicted output corresponding to a test input $x$.

Since the marginal probability $p\left( {Y{\mathrm{|}}X} \right) = {\text{ }}\int {p\left( {Y{\mathrm{|}}X,W} \right)p\left( W \right)} {\mathrm{d}}W$ in equation (1) is analytically intractable, we approximate the exact posterior distribution with a variational distribution ${q_\theta }\left( W \right).$ following the variational interpretation of dropout [34], we adopt dropout masks during training and minimizing the Kullback–Leibler divergence between ${q_\theta }\left( W \right)$ and true posterior. At test-time, we kept dropout active and performed $T$ stochastic forward passes. Each pass draws a weight sample ${W_t}\sim {q_\theta }\left( W \right)$ from the posterior [43]. This MC procedure approximate equation (2) to obtain final probability $\hat p\left( {y{\mathrm{|}}x} \right){\text{ }}$ by averaging the predicted probabilities ${p_t}\left( {y{\mathrm{|}}x} \right){\text{ }}$ across $T$ forward passes:

\begin{equation*}\hat p\left( {y{\mathrm{|}}x} \right) = \frac{1}{T}\mathop \sum \limits_{t = 1}^T {p_t}\left( {y{\mathrm{|}}x,{ }{W_t}} \right).\end{equation*}

To quantify epistemic uncertainty, we compute the normalized Shannon entropy $H\left( x \right)$ of the mean probability:

\begin{equation*}H\left( x \right) = - \frac{1}{{\log 2}}\left[ {\hat p\left( {y{\mathrm{|}}x} \right)\log \hat p\left( {y{\mathrm{|}}x} \right) + \left( {1 - \hat p\left( {y{\mathrm{|}}x} \right)} \right)\log \left( {1 - \hat p\left( {y{\mathrm{|}}x} \right)} \right)} \right].\end{equation*}

### 2.4. Calibration

To ensure the clinical applicability and reliability of the ConQuE model, we implemented a calibration procedure to determine an uncertainty threshold that meets predefined clinical sensitivity (recall rate) requirements. This threshold serves as a decision boundary to identify predictions deemed trustworthy for clinical decision-making, while flagging uncertain cases for clinician review. We hypothesized that an effective uncertainty quantification approach would yield predictions where lower uncertainty values correspond to higher recall for *revision required* cases.

To test our hypothesis and calibrate the uncertainty scores output by the ConQuE model, we first obtained the classification results (i.e. *acceptable* vs. *revision required*) and associated uncertainty estimates for each sample in the validation data cohort. The predictions were sorted in ascending order of uncertainty, and cumulative recall rate for the ‘*revision required*’ class was computed across the ordered set. This process generated the ‘recall vs. uncertainty’ calibration curve shown in figure 1, which characterizes how well uncertainty estimate captures clinically important problematic contours. Under a well-trained uncertainty estimation framework, we expect this curve to exhibit a decreasing trend, indicating lower uncertainty values are associated with higher sensitivity in detecting suboptimal contours.

Based on this calibration curve, we identified the uncertainty threshold corresponding to a predefined clinical sensitivity requirement. For instance, if clinical use demands a minimum of 98% sensitivity for automated decisions, we located the point on the calibration curve where the cumulative recall first drops below 98%. The corresponding uncertainty value was then set as the clinical uncertainty threshold for ConQuE model. During testing and model deployment phase, predictions with uncertainty values below the threshold are considered reliable and could be automatically accepted, while predictions exceeding this threshold are flagged for further review. This process enhances both the safety and efficiency of the clinical workflow. Notably, this calibration step is modular and can be repeated post-deployment using new data accumulated during routine clinical use. By periodically updating the uncertainty threshold based on fresh calibration data, the ConQuE model could remain robust to potential model drift over time.

### 2.5. Patient data

We retrospectively collected data from 311 prostate cancer patients treated on the 1.5 T MR-LINAC system (Elekta AB, Stockholm, Sweden) at our institution. For each patient, only the initial reference treatment plan was used, as its contour structure set was independently generated and not adapted from any prior plans. Patient MR scans and corresponding target organ contours were extracted for subsequent processing from these reference plans.

**Figure 3.**
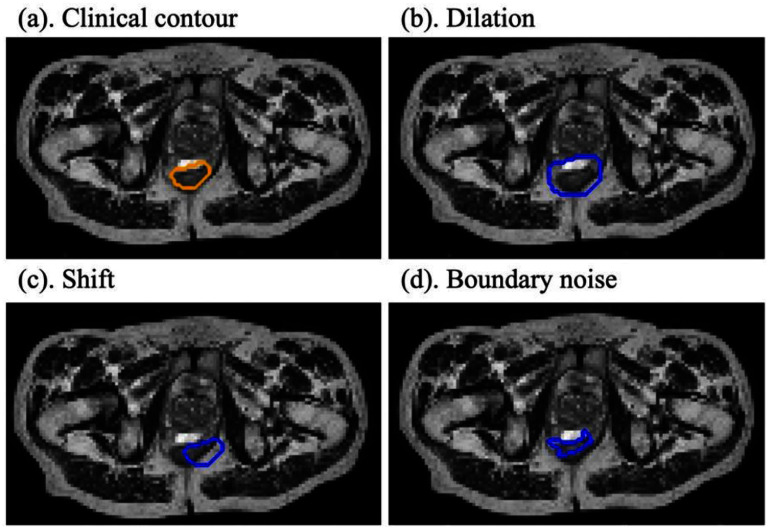
Illustration of rectum contour quality. (a) Clinically approved contour labeled as *acceptable*. (b)–(d) Synthetic *revision required* contours generated using different perturbation methods: (b) dilation, (c) spatial shift, and (d) random boundary noise.

To standardize spatial resolution across all cases, MR images were resampled to an in-plane resolution of $3\,{\mathrm{mm}} \times 3\,{\mathrm{mm}}$. *Acceptable* contours were obtained directly from the clinically approved initial reference treatment plans, which were manually delineated by expert clinicians at our institution on MR images. To simulate *revision required* contours for training and evaluation purposes, we applied controlled perturbations to the binary masks of clinically approved contours using the OpenCV Python library [44]. Four types of synthetic errors were introduced at the 2D slice level: dilation, erosion, translation, and boundary noise (figure 3). Specifically, dilation and erosion operations were performed by expanding or contracting its boundaries by approximately two pixels randomly along the *x*-axis, *y*-axis, or both directions. In addition, we also applied random shift to the entire binary mask within a range of $ \pm 2$ pixels. To ensure that a meaningful perturbation was applied, shifts of zero in both directions were explicitly excluded. Furthermore, boundary noise was introduced by first randomly selecting up to $15$ points along the contour boundary. Each selected point was then displaced in both *x*- and *y*-directions by a random integer offset within $ \pm 2$ pixels. The resulting deformed contour was then redrawn and filled to generate a new binary mask with irregularities along the boundary. This approach preserved the general shape of the organ while simulating non-uniform contouring inconsistencies. The perturbation magnitudes were intentionally kept small to maintain anatomical plausibility and avoid introducing unrealistic deformations.

We extracted paired 2D axial MR slices and their corresponding binary segmentation masks for multiple organs, including prostate (treatment target), and six OARs including rectum, urethra, bilateral femoral heads, bladder, penile bulb, and cauda equina. A detailed summary of the slice-wise distribution of *acceptable* (clinically approved) contours for each organ is provided in table 1. The dataset was split at the patient level into training (249 patients), validation (31 patients), and testing (31patients) sets to ensure no data leakage between sets. During training, synthetic *revision required* contours were dynamically generated for each mini-batch. To ensure stable learning, an equal number of *acceptable* and *revision required* contours were sampled for each organ in every training epoch.

**Table 1.** Distribution of *acceptable* contours (slices) across different organs and training splits.

Organ	Prostate	Rectum	Femur heads	Urethra	Bladder	Penile bulb	Cauda equina
Training	2094	5152	5701	3747	5118	1538	2606
Validation	224	794	678	558	580	230	572
Test	301	791	736	493	619	156	442
Total	2618	6737	7115	4798	6317	1924	3620

### 2.6. Analysis

During the testing phase, we synthetically generated an equal number of *revision-required* slices to match the number of *acceptable* testing contours in table 1 and maintain a balanced evaluation. The ConQuE model was evaluated using MC dropout with $T = 30$ stochastic forward passes per input to enable uncertainty estimation. The model’s performance in contour quality classification was assessed both overall and organ-specifically using standard evaluation metrics: accuracy, precision, recall, F1-score, and area under the receiver operating characteristic curve (AUC). With a fixed contour quality threshold of $0.5$, these metrics were computed based on binary classification of contours as either *acceptable* ($\lt\!\!0.5$) or *revision required* ($ \ge\! \!0.5$). To validate the effectiveness and reliability of uncertainty quantification method, we examined the relationship between estimated model uncertainty and two predictive performance metrics including accuracy and recall rate on the testing dataset. Our hypothesis is a well-trained model is expected to show decreasing accuracy and sensitivity as uncertainty increases. Calibration analysis was performed on the validation cohort, and organ-specific uncertainty thresholds corresponding to clinically relevant recall targets were identified to guide decision making, i.e., accept model prediction as the QA result if uncertainty below the threshold, and flag contour for review otherwise. Additionally, the inference time of the ConQuE framework under the MC dropout setting was recorded to evaluate its runtime efficiency.

## 3. Results

### 3.1. Overall performance

The average computation time for the QA system with 30 independent forward passes for MC dropout was $\sim13.1 \pm 5.18$ ms per slice. Across the 31 testing patients, the proposed ConQuE model demonstrated robust performance in evaluating segmentation contour quality across multiple organs, with an overall accuracy of $93.9{{\% }}$. Specifically, the model achieved overall precision, recall, F1-score, and AUC values of $91.4{{\% }}$, $96.9{{\% }}$, 94.1%, and $94.8{{\% }}$, respectively. At the organ-specific level, accuracy was consistently high, with values near or above 90% for all organs (table 2), indicating consistently strong predictions. The model also exhibited high recall rates across organs, demonstrating its strong capability in identifying contours that require further revision. The AUC of all OARs involved in this study were $ \ge 93{{\% }}$, suggesting our ConQuE model effectively differentiates between *acceptable* and *revision required* contours.

**Table 2.** Quantitative performance on testing cases of ConQuE across all organs. Results are reported as ${\mathrm{mean}} \pm {\text{standard deviation}}$ across 30 forward passes.

Organ	Accuracy	Recall	Precision	F1	AUC
Prostate	$90.5 \pm 0.7$	$97.3 \pm 0.9$	$85.7 \pm 0.8$	$91.1 \pm 0.6$	$94.0 \pm 0.8$
Rectum	$95.1 \pm 0.4$	$96.5 \pm 0.5$	$94.0 \pm 0.6$	$95.2 \pm 0.4$	$96.0 \pm 0.5$
Femur heads	$94.1 \pm 0.4$	$97.8 \pm 0.5$	$91.0 \pm 0.5$	$94.3 \pm 0.4$	$93.4 \pm 0.5$
Urethra	$92.0 \pm 0.5$	$98.4 \pm 0.7$	$87.2 \pm 0.5$	$92.5 \pm 0.5$	$93.1 \pm 0.6$
Bladder	$93.5 \pm 0.5$	$96.9 \pm 0.6$	$90.6 \pm 0.6$	$93.7 \pm 0.5$	$94.6 \pm 0.6$
Penile bulb	$89.7 \pm 0.7$	$96.8 \pm 0.9$	$84.8 \pm 0.8$	$90.5 \pm 0.6$	$93.6 \pm 0.8$
Cauda equina	$94.6 \pm 0.6$	$95.2 \pm 0.7$	$94.0 \pm 0.8$	$94.6 \pm 0.5$	$95.3 \pm 0.7$

### 3.2. Uncertainty quantification

**Figure 4.**
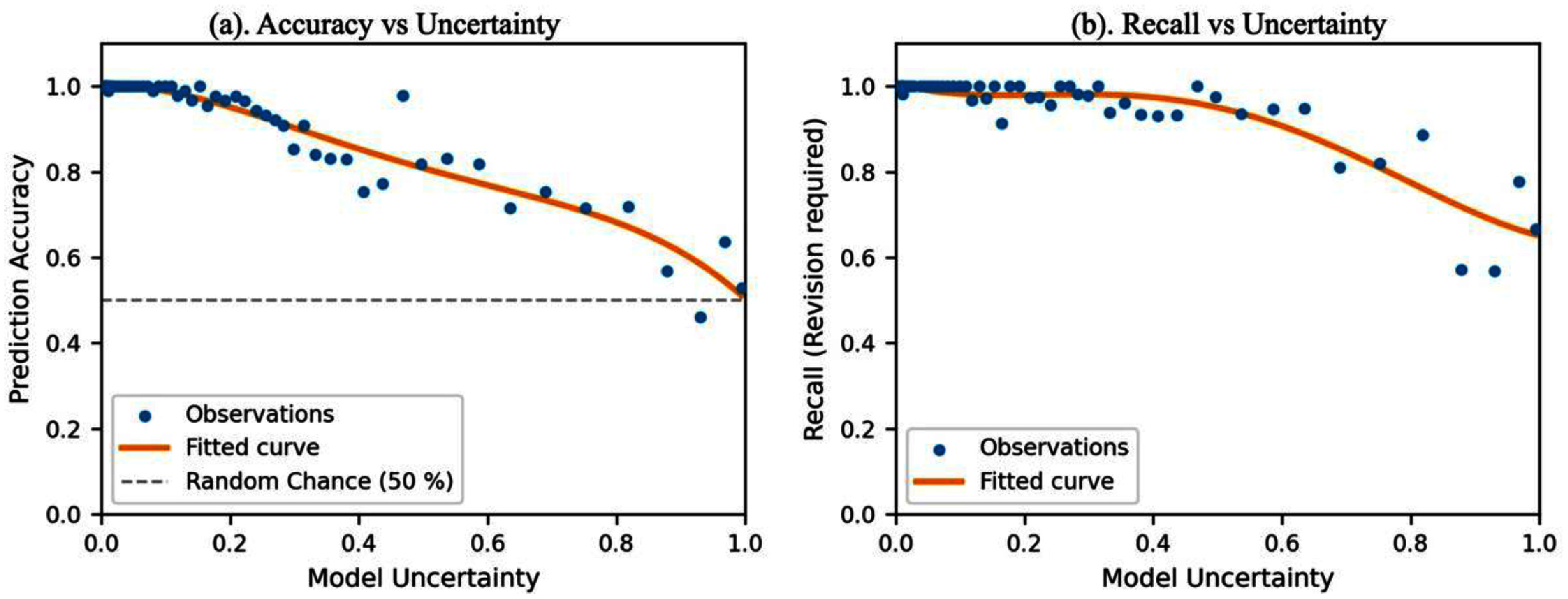
Calibration scatter plots illustrating the relationship between model prediction uncertainty and (a) overall prediction accuracy, and (b) recall rate for ‘*revision required*’ cases.

To examine the effectiveness of uncertainty quantification with MC dropout, we analyzed the relationship between model uncertainty and two predictive performance metrics including accuracy and recall rate. Ideally, an effective uncertainty estimate should exhibit a strong inverse correlation with prediction performance. As shown in figure 4, model uncertainties in testing dataset were grouped into 80 bins and performance metrics were averaged within each bin. In figure 4(a), the mean prediction accuracy was calculated within each bin. Prediction accuracy demonstrated a clear downward trend as uncertainty increased, indicating that the uncertainty scores effectively reflect the likelihood of incorrect predictions. In figure 4(b), we examined the sensitivity by evaluating recall rate for the ‘*revision required*’ class, which is particularly critical in clinical settings where missed segmentation errors can compromise treatment quality. A similar decreasing trend was observed in recall with increasing uncertainty, further supporting the effectiveness of uncertainty quantification in identifying potentially unreliable predictions.

### 3.3. Calibration

**Figure 5.**
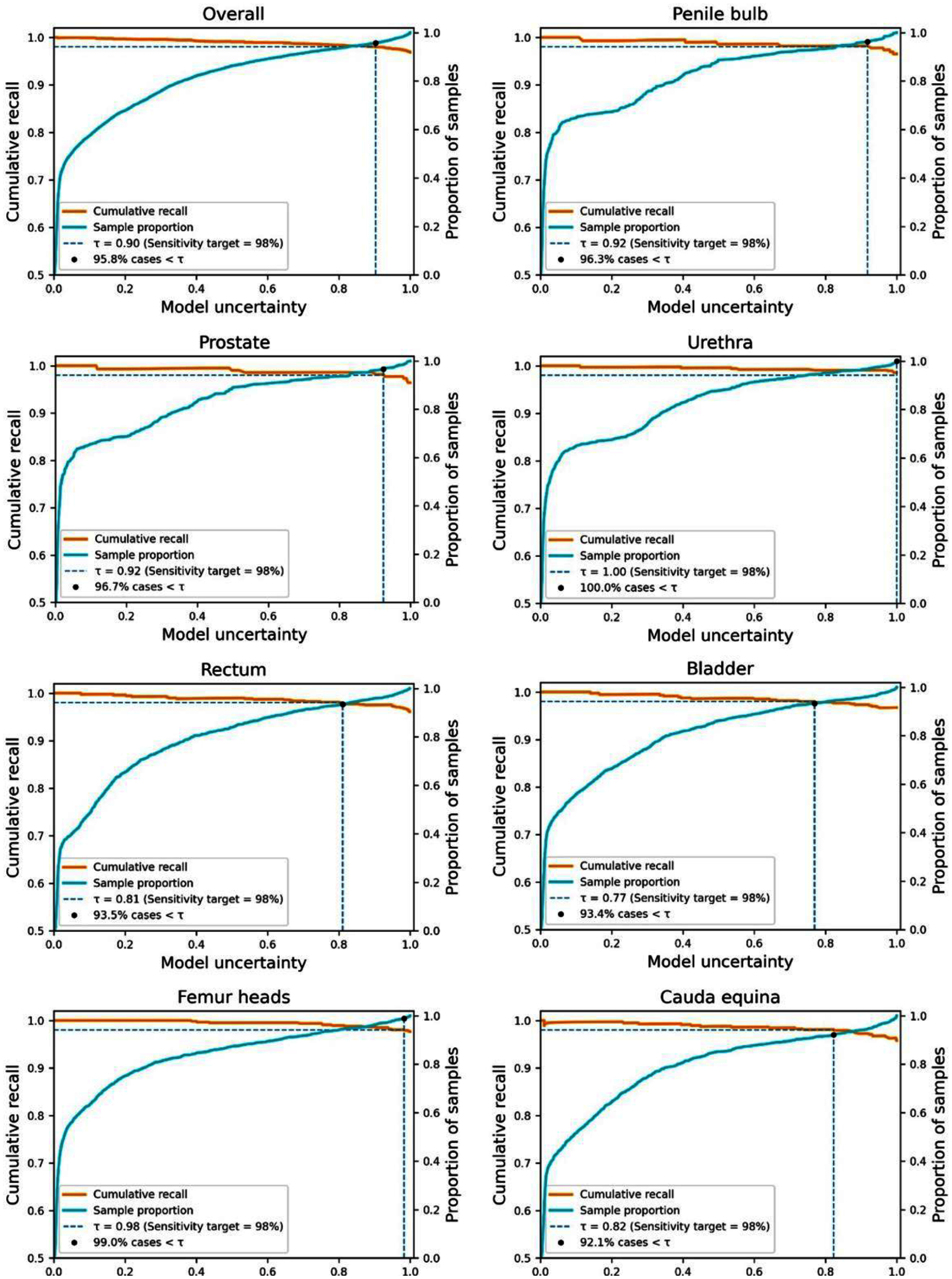
Calibration plots to determine the uncertainty threshold ($\tau )$ required to achieve clinical goal of sensitivity.

To support clinical deployment, we established uncertainty-based decision thresholds by analyzing the relationship between prediction uncertainty and sensitivity for *revision required* cases using the validation dataset. For each organ, predictions were sorted in ascending order of uncertainty, and the cumulative recall for *revision required* contours was computed. Figure 5 presents the resulting calibration plots for all organs of interest, illustrating the relationship between model-predicted uncertainty and the cumulative recall of *revision required* contours (orange curve). In addition to recall, the cumulative proportion of contour slices in test cases (cyan curve) is plotted to indicate how much of the population would be retained under a given uncertainty threshold.

Across all organs, cumulative recall generally decreases with increasing uncertainty, affirming that uncertainty estimation is effective for identifying *revision required* cases in our model. The population curves also show that a substantial proportion of evaluated cases tend to cluster in the low-uncertainty range, supporting the feasibility of selective acceptance under clinically relevant sensitivity thresholds. Structure-specific uncertainty thresholds were derived from the calibration plots shown in figure 5 by identifying the level of uncertainty that achieves a sensitivity of 98%. For instance, to achieve a recall of $ \unicode{x2A7E} $ 98% for penile bulbs, the uncertainty must be below 0.92, a threshold met by 96.3% of the evaluated samples. The threshold will serve as actionable criteria to facilitate downstream decision-making by flagging higher-uncertainty cases for manual review.

To validate this calibration process, the finalized thresholds based on validation data were applied to the independent testing dataset. More specifically, model performance was further evaluated on samples with uncertainty values below the organ-specific thresholds. Table 3 summarizes the accuracy, recall, and percentage of eligible samples for each organ. Across all organs, most contour slices in the test cases (defined as *population*) fall below the organ-specific uncertainty thresholds, demonstrating the capability of the proposed framework in improving the efficiency of contour QA. For instance, only 3.2% of QA predictions for prostate contours exceeded the corresponding uncertainty threshold and would therefore be flagged for manual review. Moreover, for those testing samples with lower uncertainty, the ConQuE model was able to achieve desired sensitivity (close to or higher than 98%) for all organs considered.

**Table 3.** Quantitative performance on test cases with uncertainty below the thresholds ($\tau )$. *Population* is defined as contour slices for each organ.

Organ	Prostate	Rectum	Femur heads	Urethra	Bladder	Penile bulb	Cauda equina
$\tau $ (recall = 98%)	0.92	0.81	0.98	1.00	0.77	0.92	0.82
Accuracy	92.1	96.7	94.6	92.0	95.7	91.9	97.9
Recall	98.6	97.9	98.2	98.4	98.1	98.6	98.0
Population ($< \tau $)	96.8%	94.7%	98.8%	100%	93.5%	94.9%	91.2%

## 4. Discussion

Despite significant advancements in AI-based segmentation models enhancing delineation efficiency in the clinical workflow, several critical limitations remain unresolved. These models often encounter difficulties accurately segmenting complex anatomical structures with inherently low image contrast and suffer performance degradation over time due to data heterogeneity resulting from varied imaging protocols. Without timely and reliable QA, undetected segmentation errors may compromise treatment planning accuracy and ultimately affect patient outcomes. Manual segmentation QA has long been the conventional approach for identifying such errors, yet it is inherently time-consuming, labor-intensive, and not well-suited for time-sensitive clinical workflows such as oART. In this context, AI-assisted automatic contour QA methods have thus emerged as a promising solution to enhance efficiency, scalability, and consistency in the routine clinical use of AI-based segmentation models. However, existing AI-based QA methods typically provide definitive quality predictions without quantifying the uncertainty or confidence of their assessments, limiting clinical trustworthiness. Furthermore, current uncertainty-based approaches primarily focus on refining segmentation accuracy by highlighting uncertain areas. These methods often provide voxel-level uncertainty maps that are difficult to interpret directly and may not translate into clinically actionable decisions, such as accepting or revising an entire contour. Additionally, most existing QA models are tailored for single-organ tasks, requiring dedicated models for individual structures, which hampers their applicability in multi-organ clinical workflows.

To overcome these limitations, we have proposed a multi-structure AI-driven QA framework integrating uncertainty quantification for efficient and robust contour QA. Our model leverages MC dropout to quantify prediction uncertainty, allowing it to generate both a QA decision (‘*acceptable*’ or ‘*revision required*’) and an associated confidence score for contours of multiple structures. Our proposed model demonstrated robust real-time performance ($\sim13.1\,{\mathrm{ms}}$ per slice), while achieving high overall accuracy of 93.9% and sensitivity of 96.9%. The effectiveness of the proposed uncertainty quantification approach was demonstrated by the observed trend that lower model uncertainty is consistently associated with improved predictive performance. This enabled the derivation of organ-specific uncertainty thresholds that satisfy clinically defined sensitivity targets supporting the use of ConQuE in a real-world deployment. By applying these thresholds obtained based on validation data to the independent testing dataset, ConQuE facilitates automation in the segmentation QA process for over 90% of testing cases with prediction uncertainty lower than the thresholds, achieving recall rates above 98% in identifying contours requiring revision. Since the prostate is the treatment target, the clinical consequences of accepting a poor-quality contour are particularly significant. To mitigate this risk, we propose adopting a stricter uncertainty threshold specifically for the prostate QA, ensuring that all questionable contours are routed to clinicians for review. With our validation data, the uncertainty threshold required to achieve sensitivity of 100% was $0.11$ for the prostate target. Applied it to the test dataset, it yielded 100% recall rate in identifying revision-required prostate contour slices. In consequence, only 66.1% of contour slices could be auto-accepted and the remaining 33.9% would require manual review. This structure-specific policy minimizes the likelihood of missed errors and safeguards treatment safety, albeit at the expense of a modest increase in review workload. These results support the potential value of ConQuE framework in improving contour QA effectiveness and efficiency, especially in oART with heavy time constraints.

While the proposed model demonstrates strong overall performance, several directions for further enhancement were identified. First, the model achieved a higher recall rate than precision for most organs, indicating that it has higher accuracy in detecting *revision required* contours compared to *acceptable* ones. While this conservative behavior could increase the number of false-positive flags and potentially add review time for clinicians, it aligns with clinical priorities, where avoiding missed revision-required contours is paramount. This conservative tendency could be partly attributed to the subjective definition of contour labeling in the training data. We plan to work with the clinicians to review contours from both pre-planning and online sessions and manually label the contours. This will provide more clinically meaningful training data. Secondly, organ-specific variations in performance were observed. Structures such as the prostate, urethra, and penile bulb yielded comparatively lower accuracy. This could be attributable to a combination of imbalanced data distribution across structures and the inherent anatomical complexities of certain organs. Similar findings have been reported in prior studies of multi-organ segmentation models [45, 46], where small organs with indistinct boundaries (e.g., urethra and penile bulb), demonstrated lower segmentation accuracy. As part of the future work, we will explore common strategies such as the synthetic minority oversampling technique [47, 48] to generate additional training samples for minority classes such as prostate, urethra, and penile bulb. In addition, we do recognize the perturbation strategies can impact organs differently depending on size and morphology, which may bias the model performance. Another potential future improvement of this model will be implementing size-aware perturbations to generate *revision required* contours for training. Finally, while the current model operates in a slice-by-slice fashion, future work will extend the model to incorporate 3D spatial context and dose information to enable more comprehensive contour quality assessment.

Moving forward, we aim to validate the proposed framework prospectively within MRgOART workflows, quantifying efficiency gains and confirming clinical safety. Clinical deployment will require seamless integration with treatment planning systems to automate data extraction of daily MR images and corresponding structure sets. We will also incorporate an active learning loop that logs clinician overrides and periodically uses this feedback to refine the model, thereby maintaining alignment with evolving clinical standards and newly observed error patterns. Demonstrating reproducible accuracy and robustness under these real-world conditions will be critical for establishing the ConQuE model as a dependable QA tool. By delivering rapid and trustworthy contour evaluation, the framework has the potential to streamline oART workflows and ultimately enhance both patient safety and overall treatment quality.

## 5. Conclusion

In summary, we proposed a multi-organ, AI-driven contour QA framework that integrates uncertainty estimation via MC dropout to deliver both quality assessments and confidence scores. The proposed ConQuE framework achieves consistently high accuracy and recall across multiple organs, with sub-second inference times suitable for clinical deployment in time-sensitive oART workflows. Calibration analyses confirmed the inverse relationship between model uncertainty and prediction performance, which enabled the establishment of an uncertainty threshold that meets predefined clinical sensitivity requirements to support reliable decision making. By offering uncertainty-informed assessments without imposing substantial computational overhead, the framework allows clinicians to focus manual review on a small subset of cases with high prediction uncertainties, thereby improving the effectiveness and efficiency of segmentation QA process in oART workflows.

## Data Availability

The data cannot be made publicly available upon publication because they contain sensitive personal information. The data that support the findings of this study are available upon reasonable request from the authors.
